# Analyzing the Transcriptomes of Two Quorum-Sensing Controlled Transcription Factors, RcsA and LrhA, Important for *Pantoea stewartii* Virulence

**DOI:** 10.1371/journal.pone.0145358

**Published:** 2015-12-23

**Authors:** Alison Kernell Burke, Duy An Duong, Roderick V. Jensen, Ann M. Stevens

**Affiliations:** Department of Biological Sciences, Virginia Tech, Blacksburg, VA 24061, United States of America; Centre National de la Recherche Scientifique, Aix-Marseille Université, FRANCE

## Abstract

The Gram-negative proteobacterium *Pantoea stewartii* subsp. *stewartii* causes wilt disease in corn plants. Wilting is primarily due to bacterial exopolysaccharide (EPS) production that blocks water transport in the xylem during the late stages of infection. EsaR, the master quorum-sensing (QS) regulator in *P*. *stewartii*, modulates EPS levels. At low cell densities EsaR represses or activates expression of a number of genes in the absence of its acyl homoserine lactone (AHL) ligand. At high cell densities, binding of AHL inactivates EsaR leading to derepression or deactivation of its direct targets. Two of these direct targets are the key transcription regulators RcsA and LrhA, which in turn control EPS production and surface motility/adhesion, respectively. In this study, RNA-Seq was used to further examine the physiological impact of deleting the genes encoding these two second-tier regulators. Quantitative reverse transcription PCR (qRT-PCR) was used to validate the regulation observed in the RNA-Seq data. A GFP transcriptional fusion reporter confirmed the existence of a regulatory feedback loop in the system between LrhA and RcsA. Plant virulence assays carried out with *rcsA* and *lrhA* deletion and complementation strains demonstrated that both transcription factors play roles during establishment of wilt disease in corn. These efforts further define the hierarchy of the QS-regulated network controlling plant virulence in *P*. *stewartii*.

## Introduction

*Pantoea stewartii* subsp. *stewartii* (*P*. *stewartii*) is a Gram-negative rod-shaped, gamma-proteobacterium that belongs to the *Enterobacteriaceae* family containing plant-associate enterics (eg. *Erwinia*, *Dickeya*, and *Pectobacterium* spp.) and human enteric pathogens associated with plants (eg. *Escherichia* and *Salmonella* spp.). *P*. *stewartii* is the causative agent of Stewart’s wilt in maize [1]. It is transmitted passively from the gut of the corn flea beetle, *Chaetocnema pulicaria*, to the plant leaves via the feeding process of the insect vector [2]. The symptoms of the disease include water-soaked lesions in the leaf when the bacterium is in the apoplast during the early stages of infection and wilt due to biofilm formation in the xylem during the later stages of infection; ultimately this can lead to plant death [3]. The virulence of the bacterium is controlled through quorum sensing (QS) [4], a cell-to-cell communication system found primarily in eubacteria.

Gamma-proteobacteria, like *P*. *stewartii*, most commonly produce N-acyl homoserine lactone (AHL) signals during QS due to the activity of a LuxI-type protein. This intercellular signal then interacts with the master regulatory protein in the bacterium, a LuxR-type protein, to coordinate global gene expression. A variety of physiological outputs are controlled by QS such as bioluminescence, biofilm formation, virulence factor expression or exoenzyme production [5–9]. In *P*. stewartii, the QS signal N-3-oxo-hexanoyl-L-homoserine lactone (3-oxo-C_6_-HSL) is synthesized by the AHL synthase EsaI, a LuxI homologue [10]. At low cell density, the master QS regulatory protein EsaR, a LuxR homologue, is active and binds its recognition sites in the DNA to repress or activate transcription of different gene targets [10–12]. However, when EsaR and AHL form a complex, EsaR becomes inactive and unable to bind to DNA resulting in derepression or deactivation of target gene transcription at high cell density [13]. Several direct targets of EsaR have been identified through classic genetic [4], proteome-level [14] and transcriptome-level [15] analysis. Two of these direct targets, *rcsA* and *lrhA*, encode transcription factors elucidating additional levels of downstream regulation in response to QS.

RcsA is a transcription factor in the Rcs (regulation of capsule synthesis) regulatory network involved in colanic acid and K antigen capsular polysaccharide synthesis (cps) in *Escherichia coli* [16, 17] and *Salmonella typhi* [18]. It has been established that a major virulence factor of *P*. *stewartii*, the exopolysaccharide (EPS), is controlled via direct EsaR-mediated repression of *rcsA* (CKS_2570) [4]. Avirulent strains lacking EPS production typically have disruptions in genes found in either *rcsA* or the *cps* locus [4]. In *P*. *stewartii*, RcsA (in conjunction with RcsB) has been proposed to activate three *cps/wce* gene clusters, comprised of (I) *wceG1*, *wza*, *wzb*, *wzc*, *wceL*, *wceB*, *wceM*, *wceN*, *wceF*, *wceJ*, *wceK*, *wzx*, *galF*, and *galE*, (II) *wceG2* and (III) *wceO and wzx* [4, 19]. However, the complete RcsA regulon in *P*. *stewartii* has not been fully defined.

The LysR-type regulator LrhA (CKS_2075) is directly activated by EsaR in *P*. *stewartii* [14, 15]. In *E*. *coli*, LrhA indirectly controls expression of flagella, motility and chemotaxis by positively autoregulating its own expression and repressing the synthesis of the master regulator of flagella and chemotaxis gene expression, the FlhD_2_C_2_ heterotetramer, thereby suppressing motility and chemotaxis [20]. *P*. *stewartii* possesses swarming rather than swimming motility, and swarming motility is critical to the pathogenicity of the bacterium [21]. QS controls this motility in an interesting and complicated manner; a lack of AHL inhibits the motility, while lacking both AHL and EsaR attenuates swarming. It has been proposed that QS indirectly controls motility via stewartan synthesis regulated by the RcsA/B phosphorelay system [21]. Expression of the FlhD_2_C_2_ flagellar master regulator is directly control by the RcsA/B system in other bacteria [22–24]. However, the precise role of LrhA in *P*. *stewartii* and its relationship to both motility and RcsA is largely undefined.

To elucidate the downstream roles of RcsA and LrhA, phenotypic assays, RNA-Seq, qRT-PCR, GFP assays, and *in planta* virulence assays were used to evaluate the differences between the *rcsA* and *lrhA* deletion strains in comparison to the wild type. The identification of the most positively and negatively regulated targets for these two key downstream transcription factors provides greater insight into the coordinated regulation of genes in the QS network.

## Materials and Methods

### Strains and growth conditions

Strains and plasmids utilized in this study are listed in Table 1. *E*. *coli* strains were grown in Luria-Bertani (LB) (10 g/L tryptone, 5 g/L yeast extract, and 5 g/L NaCl) broth or on plates with 1.5% agar and *P*. *stewartii* strains were grown in either LB or Rich Minimal (RM) medium (1X M9 salts, 2% casamino acids, 1 mM MgCl_2_, and 0.4% glucose). The growth medium was supplemented with nalidixic acid (Nal, 30 μg/ml), ampicillin (Ap, 100 μg/ml), kanamycin (Kn, 50 μg/ml), chloramphenicol (Cm, 30 μg/ml), or streptomycin (Str, 100 μg/ml) as required (see Table 1). *P*. *stewartii* strains were grown at 30°C, while *E*. *coli* strains were maintained at 37°C.

**Table 1:** Strains and plasmids used in the study.

Strains	Genotype and notes^a^	References
***Pantoea stewartii* strains**		
DC283	Wild-type strain; Nal^r^	[26]
*ΔlrhA*	Unmarked deletion of *lrhA* coding sequence; Nal^r^	This study
Δ*lrhA/lrhA* ^*+*^	DC283 *ΔlrhA* with chromosomal complementation of *lrhA* and its promoter downstream of *glmS*; Nal^r^ Cm^r^	This study
Δ*rcsA*	Unmarked deletion of *rcsA* coding sequence; Nal^r^	This study
Δ*rcsA/rcsA* ^+^	DC283 *ΔrcsA* with chromosomal complementation of *rcsA* and its promoter downstream of *glmS*; Nal^r^ Cm^r^	This study
***Escherichia coli* strains**		
Top 10	F^—^ *mcrA* Δ(*mrr-hsdRMS-mcrBC*) *Φ80dlacZΔM15* Δ*lac*X74 *deoR recAI araD139* Δ(*ara-leu)7697 galU galK rpsL (*Str^r^ *) endA1 nupG*	[45]
DH5α	*F- endA1 glnV44 thi-1 recA1 relA1 gyrA96 deoR nupG Φ80dlacZΔM15 Δ(lacZYA-argF)U169*, *hsdR17(rK- mK+)*	[46]
DH5α *λpir*	*F- endA1 glnV44 thi-1 recA1 relA1 gyrA96 deoR nupG Φ80dlacZΔM15 Δ(lacZYA-argF)U169*, *hsdR17(rK- mK+)*, *λpir*	[25]
CC118 *λpir*	*Δ(ara-leu)*, *araD*, *ΔlacX74*, *galE*, *galK*, *phoA20*, *thi-1*, *rpsE*, *rpoB*, *argE(Am)*, *recA1*, *λpir*	[27]
S17-1 *λpir*	*recA pro hsdR RP4-2-Tc*::*Mu-Km*::*Tn7*	[31]
**Plasmids**		
pGEM-T	Cloning vector, Ap^r^	Promega
pDONR201	Entry vector in the Gateway system, Kn^r^	Life Technologies
pAUC40	Suicide vector pKNG101::*attR*-*ccdB*-Cm^R^; Cm^r^, Str^r^, *sacB*	[19]
pEVS104	Conjugative helper plasmid, *tra trb;* Kn^r^	[28]
pUC18R6K-mini-Tn7-cat	Tn7 vector for chromosomal integration into the intergenic region downstream of *glmS*; Cm^r^, Ap^r^	[30]
pPROBE’GFP[tagless] P_*rcsA*_	pPROBE’GFP[tagless] vector with the promoter of *rcsA;* Kn^r^	This study

^*a*^ Ap^**r**^, ampicillin resistance; Nal^**r**^, nalidixic acid resistance; Kn^r^, kanamacyin resistance; Gm^r^, gentamycin resistance; Cm^r^, chloramphenicol resistance; Str^r^, streptomycin resistance

### Construction of markerless deletion mutant strains

Chromosomal deletions of *lrhA* and *rcsA* were constructed based on the Gateway system (Life Technologies, Grand Island, NY) and suicide vectors. Two 1kb fragments from upstream and downstream of the desired deletion region were first separately amplified using the primers designated in S1 Table. After that, a two-step PCR reaction was performed in which the upstream and downstream segments for the specific gene were joined together and primers 1kbUPF-attB1 and 1kbDNR-attB2 (S1 Table), specific to each construct, were added to facilitate the Gateway BP reaction (Life Technologies). The final PCR products were cloned into the pGEM-T vector (Promega, Madison, WI) for sequencing before being transferred to the Gateway plasmids using BP Clonase II enzyme mix and then to the suicide vector pAUC40 [19] using LR Clonase II enzyme mix (Life Technologies). The resulting plasmids were transformed into competent *E*. *coli* DH5α *λ pir* cells [25]. A tri-partite conjugation was used to transfer the suicide vector constructs into *P*. *stewartii* DC283 [26] using *E*. *coli* strain CC118*λpir* [27] carrying the conjugative helper plasmid pEVS104 [28] to facilitate suicide vector transfer. Selection for the first recombination event into the *P*. *stewartii* chromosome was carried out on LB agar supplemented with Nal and Str. Then, the recombinants were plated on LB (no salt) agar supplemented with 5% sucrose [29] to select for the desired double cross-over based on SacB activity. A screen for deletion strains was performed with colony PCR using a 3-primer reaction with primers for each gene corresponding to sites upstream, downstream, and inside the specific gene of interest (S1 Table). DNA sequencing of appropriate PCR products was used to confirm the final deletion strains.

### Construction of chromosomal complementation strains

Complementation strains were constructed by generating a chromosomal insertion of the promoter and coding regions of the target gene into the neutral region downstream of *glmS* on the *P*. *stewartii* chromosome using the pUC18R6K-mini-Tn7-cat vector system developed by Choi *et al* [30]. Specifically, primer sets with either *Eco*RI and *Xho*I or *Sac*I and *Spe*I sites (S1 Table) were used to amplify the target regions and clone them into pGEM-T (Promega) for sequencing confirmation. DNA fragments of interest were moved into the pUC18R6K-mini-Tn7-cat vector using double digestion with *Eco*RI and *Xho*I or *Sac*I and *Spe*I (New England BioLabs (NEB), Ipswich, MA), followed by ligation with T4 DNA ligase (NEB) and transformation into DH5α *λpir* or S17-1 *λpir* [31]. These transformants served as the donor in the conjugation process with the appropriate deletion strain of *P*. *stewartii* as the recipient. Colony PCR reactions using one primer in the inserted gene and two primers flanking the *glmS* region [32] were conducted to screen for the presence of the chromosomal insertion. DNA sequencing of appropriate PCR products was used to validate the integrity of the complementation strains.

### Phenotypic capsule production assay

Wild-type, Δ*rcsA* and Δ*rcsA/rcsA* ^*+*^ strains were grown in LB supplemented with the appropriate antibiotics overnight at 30°C with shaking. The overnight cultures were used to inoculate fresh LB to an OD_600_ of 0.05 and grown at 30°C to OD_600_ of 0.2. The strains were then cross streaked on agar plates containing 0.1% casamino acids, 1% peptone, 1% glucose (CPG) and 1.5% agar [12]. Capsule production was assessed qualitatively after 48 hours of incubation at 30°C by visually comparing the surface appearance of the streaks.

### Phenotypic surface motility assay

Swarming motility for the wild-type, Δ*lrhA*, and Δ*lrhA/lrhA* ^*+*^ strains was investigated under strict conditions to ensure a reproducible phenotype. Overnight cultures were diluted to an OD_600_ of 0.05 in LB broth and grown to an OD_600_ of 0.5. Five μl of cell culture at OD_600_ of 0.5 were spotted directly onto the agar surface of LB 0.4% agar quadrant plates supplemented with 0.4% glucose [21], which were poured on the same day of the experiment. Plates were put at room temperature for 30 min to 1 hour before incubating them lid-up in a closed box with a flat bottom inside the 30^°^C incubator. Pictures of the plates were taken after 48 hours of incubation.

### Transcriptome analysis methods

The RNA-Seq method for analyzing the transcriptome of the wild-type *P*. *stewartii* DC283 strain and the two strains each carrying a deletion of one of the two genes, *lrhA* or *rcsA*, has been previously published [15]. Briefly, RNA was extracted from duplicate samples of each strain separately grown in RM medium using a Qiagen (Valencia, CA) miRNeasy RNA extraction kit. The total bacterial RNA was sent to the Virginia Bioinformatics Institute (VBI) (Virginia Tech, Blacksburg, VA) for Bioanalyzer quality analysis to insure RIN values greater than 9. The rRNA was depleted with an Epicentre (Madison, WI) Ribo-Zero Gram-negative Depletion Kit prior to Illumina (San Diego, CA) cDNA conversion and Illumina sequencing with single 50 bp reads.

### RNA-Seq Data analysis and qRT-PCR validation

Data in the form of fastq files was received and aligned to the *P*. *stewartii* DC283 version 8 draft genome from NCBI (NZ_AHIE00000000.1) using the Geneious 7.0 software with the default “low sensitivity” settings, which also counted the numbers of reads aligning to each protein coding gene (excluding the highly repeated genes annotated as “transposase” or “IS66 ORF2 family protein”). Microsoft Excel was then used to compute an expression level for each gene by simply normalizing the read counts for each gene to the total number of mapped reads per million mapped reads (RPM) for each sample. Then the ratio of the RPM expression values (averaged over the two biological replicates) was used to compare the wild-type and deletion strain gene expression levels. (The raw read counts and RPM normalized expression levels for each of the four samples are available in the NCBI GEO database: Accession # GSE69064.) This analysis was used to select genes for qRT-PCR confirmation and validation of the RNA-Seq data. The validation genes were required to have (1) a greater than a four-fold change in average expression (RPM) between the two strains (wild-type and deletion), (2) greater than 100 reads mapped to the reference coding sequence in at least one of the samples and (3) reproducible levels of expression (< two fold change) in duplicate trials. The Bioconductor R software package “DESeq” [33] was also used to analyze the raw read counts using a more sophisticated gene expression normalization and error model to calculate multiple testing adjusted pvalues to estimate the statistical significance of detected gene expression changes. The fold changes (DESeq foldchange) determined by this second method were very similar to our Microsoft Excel analysis for the genes with 4-fold or greater change and the adjusted pvalues (DESeq padj) for those genes selected for qRT-PCR validation were all less than 0.012 (Tables 2 and 3).

**Table 2:** List of genes differentially expressed 4-fold or more in the Δ*rcsA* RNA-Seq data.

Accession #	Locus_tag	GeneID	Product	RPM Fold Change	DESeq Fold Change	DESeq padj
**Activated by RcsA**						
ACV-0288878	CKS_4672		secreted protein	17.69	16.08	1.2E-01
ACV-0289544	CKS_2241	*wceG1*	undecaprenyl-phosphate UDP-galactose phosphotransferase	12.60	11.89	2.1E-15
ACV-0290198	CKS_2799	*osmB*	lipoprotein	9.12	8.08	1.0E+00
ACV-0286879	CKS_2708	*wceG2*	undecaprenyl-phosphate UDP-galactose phosphotransferase	7.78	7.47	2.7E-08
ACV-0288877	CKS_4671		putative outer membrane lipoprotein	7.44	6.99	5.2E-09
ACV-0289541	CKS_2244	*wza*	polysaccharide export protein	6.26	5.75	1.2E-02
ACV-0288876	CKS_4670		YmcB family protein	5.79	5.53	3.0E-07
ACV-0289540	CKS_2245	*wzb*	phosphotyrosine-protein phosphatase	5.16	4.74	6.2E-02
ACV-0289539	CKS_2246	*wzc*	tyrosine-protein kinase	5.13	4.70	2.8E-02
ACV-0288191	CKS_4022	*wceO*	beta-16-glucosyltransferase	4.75	4.46	6.3E-06
ACV-0289534	CKS_2251	*wceF*	exopolysaccharide biosynthesis protein	4.11	3.78	9.7E-02
**Repressed by RcsA**						
ACV-0290191	CKS_2806		putative formate dehydrogenase oxidoreductase protein	50.38	54.64	5.7E-34
ACV-0289299	CKS_3504		cytosine/purine/uracil/thiamine/allantoin permease family protein	8.85	9.44	1.3E-11
ACV-0289953	CKS_1065	*argC*	N-acetyl-gamma-glutamylphosphate reductase	5.25	5.50	8.6E-08
ACV-0289954	CKS_1064	*argB*	acetylglutamate kinase	5.14	5.46	2.3E-07
ACV-0291072	CKS_1283	*cysD*	sulfate adenylyltransferase subunit 2	5.07	5.57	3.0E-07
ACV-0291071	CKS_1282	*cysN*	sulfate adenylyltransferase subunit 1	4.29	4.64	4.7E-06
ACV-0289145	CKS_4942	*argI*	ornithine carbamoyltransferase 1	4.20	4.54	1.1E-05

**Table 3:** List of genes differentially expressed 4-fold or more in the Δ*lrhA* RNA-Seq data.

Accession #	Locus_tag	GeneID	Product	RPM Fold Change	DESeq Fold Change	DESeq padj
**Activated by LrhA**						
ACV-0289574	CKS_2211		hypothetical protein	7.45	6.97	7.9E-04
ACV-0288191	CKS_4022	*wceO*	beta-16-glucosyltransferase	7.43	6.80	3.7E-11
ACV-0291275	CKS_3793		cytochrome d ubiquinol oxidase subunit I	4.72	4.30	3.4E-05
**Repressed by LrhA**						
ACV-0287751	CKS_5211		putative alpha/beta superfamily hydrolase/acyltransferase	58.54	61.87	8.5E-13
ACV-0287748	CKS_5208		rhamnosyltransferase I subunit B	17.02	18.57	2.0E-05
ACV-0290189	CKS_2808		hypothetical protein	15.00	16.26	3.5E-16
ACV-0285926	CKS_2106		hypothetical protein	11.06	11.98	5.4E-17
ACV-0285999	CKS_2612		phage holin	8.80	9.15	3.5E-14
ACV-0290526	CKS_0458		putative fimbrial subunit	8.39	9.32	1.1E-09
ACV-0286015	CKS_2628		hypothetical protein	6.69	7.14	2.9E-09
ACV-0286005	CKS_2618		hypothetical protein	6.46	6.83	3.4E-04
ACV-0286003	CKS_2616		phage protein	5.73	5.92	5.4E-04
ACV-0286012	CKS_2625		hypothetical protein	5.58	5.79	9.5E-03
ACV-0286019	CKS_2632		hypothetical protein	5.53	5.67	3.3E-02
ACV-0286011	CKS_2624		hypothetical protein	5.51	5.77	1.3E-07
ACV-0286029	CKS_2642		hypothetical protein	5.46	5.63	1.1E-04
ACV-0286016	CKS_2629		phage tail sheath protein FI	5.14	5.49	6.4E-09
ACV-0290525	CKS_0459		putative fimbrial subunit	5.02	5.57	2.1E-08
ACV-0290434	CKS_0551		carbonic anhydrase	4.84	5.25	5.9E-08
ACV-0286009	CKS_2622		hypothetical protein	4.77	5.17	3.6E-03
ACV-0286013	CKS_2626		hypothetical protein	4.29	4.60	3.4E-05
ACV-0286031	CKS_2644		hypothetical protein	4.29	4.57	3.5E-04
ACV-0286018	CKS_2631		hypothetical protein	4.27	4.44	1.4E-01
ACV-0286014	CKS_2627		hypothetical protein	4.26	4.47	7.7E-03
ACV-0286028	CKS_2641		phage baseplate assembly protein V	4.17	4.55	1.4E-02
ACV-0286022	CKS_2635		hypothetical protein	4.00	4.14	3.4E-05

The qRT-PCR method used for RNA-Seq validation has been previously described in Ramachandran et al [15]. Briefly, each strain was grown in the same manner as for RNA-Seq. RNA was extracted using a miRNeasy RNA extraction kit (Qiagen) and converted to cDNA using the ABI High Capacity cDNA Reverse Transcription kit (Thermo Fisher Scientific, Waltham, MA). Primers (S2 Table) designed using Primer Express software (ABI) were optimized and used to amplify ~100 bp regions of each gene of interest to determine the abundance of each transcript. The Pfaffl method was used to compare the wild type versus mutant abundance of a transcript to determine the fold regulation [34].

### GFP fusion construction and testing

A transcriptional fusion between the *rcsA* promoter and the gene for green fluorescent protein (GFP) was created using traditional molecular techniques. The *rcsA* promoter is located within the region -600 bp upstream of the annotated translation initiation codon [35]. The restriction sites *Eco*RI and *Kpn*I were added to the 5’ and 3’ ends of the promoter sequence, respectively, through the PCR primers (S1 Table). The PCR-amplified promoter fragment was ligated into pGEM-T (Promega) and sequenced. After restriction digestion of the pGEM-T construct and pPROBE’-GFP- [tagless] vector [36], a ligation produced the final pPROBE’-GFP-[tagless] vector containing the *rcsA* promoter. *E*. *coli* DH5α was transformed with this plasmid construct which was then moved into wild-type *P*. *stewartii* DC283 via conjugation using a triparental mating with the pEVS104 helper plasmid. The conjugation plates were scanned using a Typhoon Trio Scanner (GE Healthcare, Pittsburgh, PA) set to use the blue laser to screen for GFP production. Subsequently, the Δ*lrhA* and Δ*lrhA/lrhA* ^*+*^ strains were conjugated to receive the same P_*rcsA*_ pPROBE’-GFP-[tagless] vector creating the desired reporter strains.

The transconjugates were grown in RM medium supplemented with Nal and Kn, overnight to an OD_600_ < 0.5 and then diluted in fresh RM to an OD_600_ of 0.025. The cultures were allowed to grow at 30°C with shaking at 250 RPM to an OD_600_ 0.5. The GFP production was monitored in 96-well plates using a Tecan Infinite 200 (Durham, NC) set with 485 excitation and 535 emission filters. The three strains were each analyzed in triplicate for one experiment, and the three wells were averaged together to establish the mean fluorescence. The average fluorescence reading from the blank was subtracted from the average fluorescence of each strain tested to remove background signal due to the medium. The normalized fluorescence was then divided by the OD_600_ for the sample to yield relative fluorescence readings/ OD_600_. The three average relative fluorescence readings/ OD_600_ from three different experiments were averaged together and the overall standard error and two-tailed homoscedastic Student’s t-test values calculated.

### Plant virulence assay

The procedure for conducting the virulence assays with *P*. *stewartii* strains in *Zea mays* seedlings was adapted from von Bodman et al. [12] with some modifications. Sweet corn seedlings (*Zea mays* cv. Jubilee, HPS Seed, Randolph, WI) were grown in Sunshine mix #1 soil in an growth chamber (Percival Scientific, Inc., Boone, IA) at 28°C, 80% relative humidity, 16 hours light and eight hours dark cycle, and at least 200 mE m^-2^ s^-1^ light intensity. Seedlings were inoculated seven days after planting with five μl of bacterial culture grown to an OD_600_ of 0.2 in LB broth. Cells were washed and resuspended in an equal volume of phosphate buffered saline (PBS; 137 mM NaCl, 2.7 mM KCl, 10 mM Na_2_HPO_4_ and 2 mM KH_2_PO_4_, pH 7.4) prior to plant inoculation. An incision ~1 cm long was made ~1 cm above the soil line in the stem using a sterile needle (26 G 5/8, 15.9 mm, SUB-Q, Becton, Dickinson and Company, US). Then, the bacterial suspension was inoculated into the wound by moving the pipette tip across the wound five times. Fifteen germinated plants at day seven with two separate leaves and between 6–10 cm of height were inoculated for each bacterial strain tested. The plants were observed every other day after inoculation for up to 12 days post-infection to assess the virulence by two independent observers. Disease symptom severity was scored based on an arbitrary scale of five points, in which 0 = no symptoms; 1 = few scattered lesions; 2 = scattered water soaking symptoms; 3 = numerous lesions and slight wilting; 4 = moderately severe wilt; 5 = death. Both scores for each of the 15 plants under each treatment were averaged and then the data for each treatment were averaged together and used to calculate mean and standard error across the 15 plants.

### Accession numbers

The read data for the pairs of duplicate samples for the *P*. *stewartii* wild-type, Δ*rcsA*, and Δ*lrhA* strains, have been deposited in the NCBI Sequence Read Archive (SRA) with accession numbers, GSM1691841, GSM1691842, GSM1691843, GSM1691844, GSM1691845 and GSM1691846, respectively. An Excel file summarizing the differential gene expression in total counts and normalized reads per million (RPM), using the *P*. *stewartii* DC283 version 8 NCBI gene annotations, has been deposited in the NCBI Gene Expression Omnibus (GEO) database (GEO Accession GSE69064).

## Results

### Deletion of *rcsA* or *lrhA* impacts phenotypic outputs

**Fig 1:**
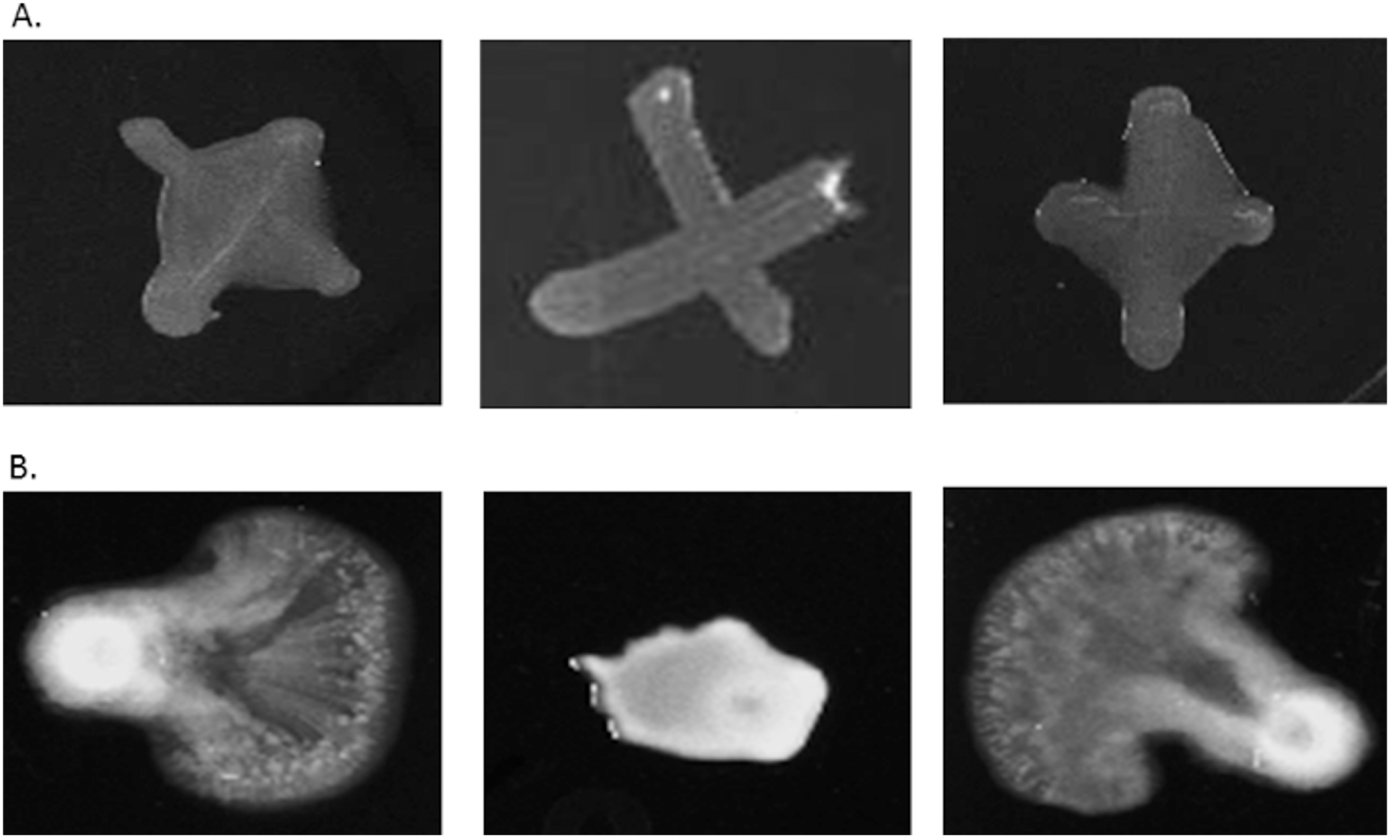
Impact of RcsA and LrhA on phenotype of *P*. *stewartii*. Panel A shows an analysis of capsule production in *P*. *stewartti* DC283 wild-type, Δ*rcsA* mutant and Δ*rcsA/rcsA* ^*+*^ complementation strains (left to right). Differences in capsule production are apparent in the regions between the arms of the X-cross streak. Panel B shows an analysis of swarming motility in wild-type, Δ*lrhA* mutant and Δ*lrhA/lrhA* ^*+*^ complementation strains (left to right). All pictures for panel A or B, respectively, were taken at the same magnification after 48 hours of incubation.

Cross streaks of the *P*. *stewartii* wild-type, Δ*rcsA* and Δ*rcsA/rcsA* ^*+*^ strains on CPG agar demonstrated that deletion of *rcsA* yielded an easily visible decrease in the level of capsule production (Fig 1A). Chromosomal complementation of *rcsA* restored capsule synthesis to levels similar to the wild type (Fig 1A).

Separately, the *P*. *stewartii* wild-type, Δ*lrhA* and Δ*lrhA/lrhA* ^*+*^ strains were analyzed for motility. Under the test conditions employed, the wild-type strain exhibited unidirectional expansion the majority of the time as described by Herrera *et al* [21] (Fig 1B), but sometimes a more symmetrical expansion was observed. The Δ*lrhA* strain exhibited a different phenotype with a noticeably smaller occupied surface area (Fig 1B). Two chromosomally complemented strains of the Δ*lrhA* strain were constructed because a smaller promoter region (~600 bp upstream of *lrhA* coding region) strain could not complement the deletion (data not shown) whereas the strain with almost the entire intergenic region upstream of *lrhA* gene (~921 bp upstream) could (Fig 1B). This suggests that there are critical regulatory elements more than 600 bp upstream in the *lrhA* promoter region.

### RNA-Seq analysis reveals the RcsA and LrhA regulons

**Fig 2:**
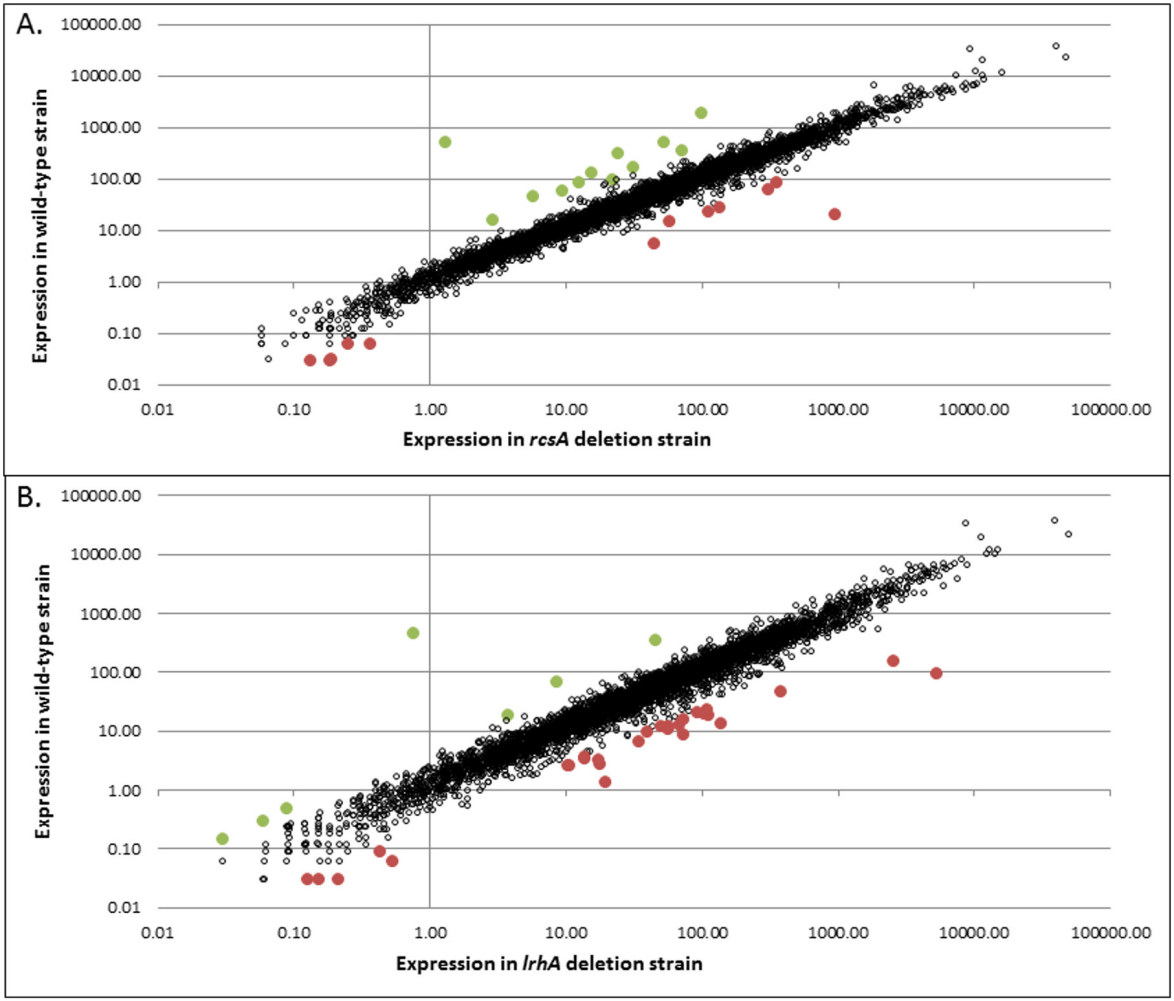
Differential mRNA expression during QS. Whole transcriptome data of the *P*. *stewartii* DC283 wild-type strain compared to the Δ*rcsA* strain (panel A) or the Δ*lrhA* strain (panel B). An open circle is used to represent each gene. Those filled with green are activated (levels of expression are four-fold or lower in the deletion strain) and those filled with red are repressed (levels of expression are four-fold or higher in the deletion strain), by either RcsA (panel A) or LrhA (panel B). The two extreme outlier points, with RPM expression >100 in the wild-type and ~1 in the deletion strains, represent the deleted genes *rcsA* or *lrhA*, respectively. All of the other colored points with RPM expression >1 in both samples are tabulated in Tables 2 and 3. The RPM change of normalized expression for most genes fall tightly around a line of slope of 1, indicating that they are approximately equally expressed in both strains.

RNA-Seq data was acquired in duplicate for the *P*. *stewartii* wild-type, Δ*rcsA*, and Δ*lrhA* strains to analyze the global impact of the two regulators. Each strain yielded 17 to 19 million reads that were mapped to the protein coding genes on the *P*. *stewartii* DC283 genome (NZ_AHIE00000000.1). The normalized gene expression data (RPM) from the two trials was averaged and then the deletion strains were compared to the wild-type strain to determine the changes in gene expression when each transcription regulator was absent (Fig 2). Genes with decreased expression in the deletion strains were considered to be positively regulated, either directly or indirectly, by RcsA or LrhA, respectively, in the wild-type strain. Conversely, genes with enhanced expression in the deletion strains were considered to be negatively regulated by the presence of LrhA or RcsA in the wild-type strain. A conservative four-fold change in RPM gene expression level was used as a selection criterion for genes to be further analyzed. An additional DESeq analysis supported the initial RPM data analysis. RcsA activates 11 genes and represses seven genes four-fold or greater (Table 2) and LrhA activates three genes and represses 23 genes four-fold or greater (Table 3). The most highly regulated RcsA-regulated genes are primarily related to capsule production, an activity previously shown to be under RcsA control [4]. The most highly regulated genes in the LrhA regulon are mostly hypothetical proteins or proteins with just putative gene function. Interestingly, a 3.04-fold repression of *rcsA* by LrhA was detected during the RNA-Seq analysis hinting at a possible coordination of regulation between RcsA and LrhA.

### qRT-PCR validates the RNA-Seq data

**Fig 3:**
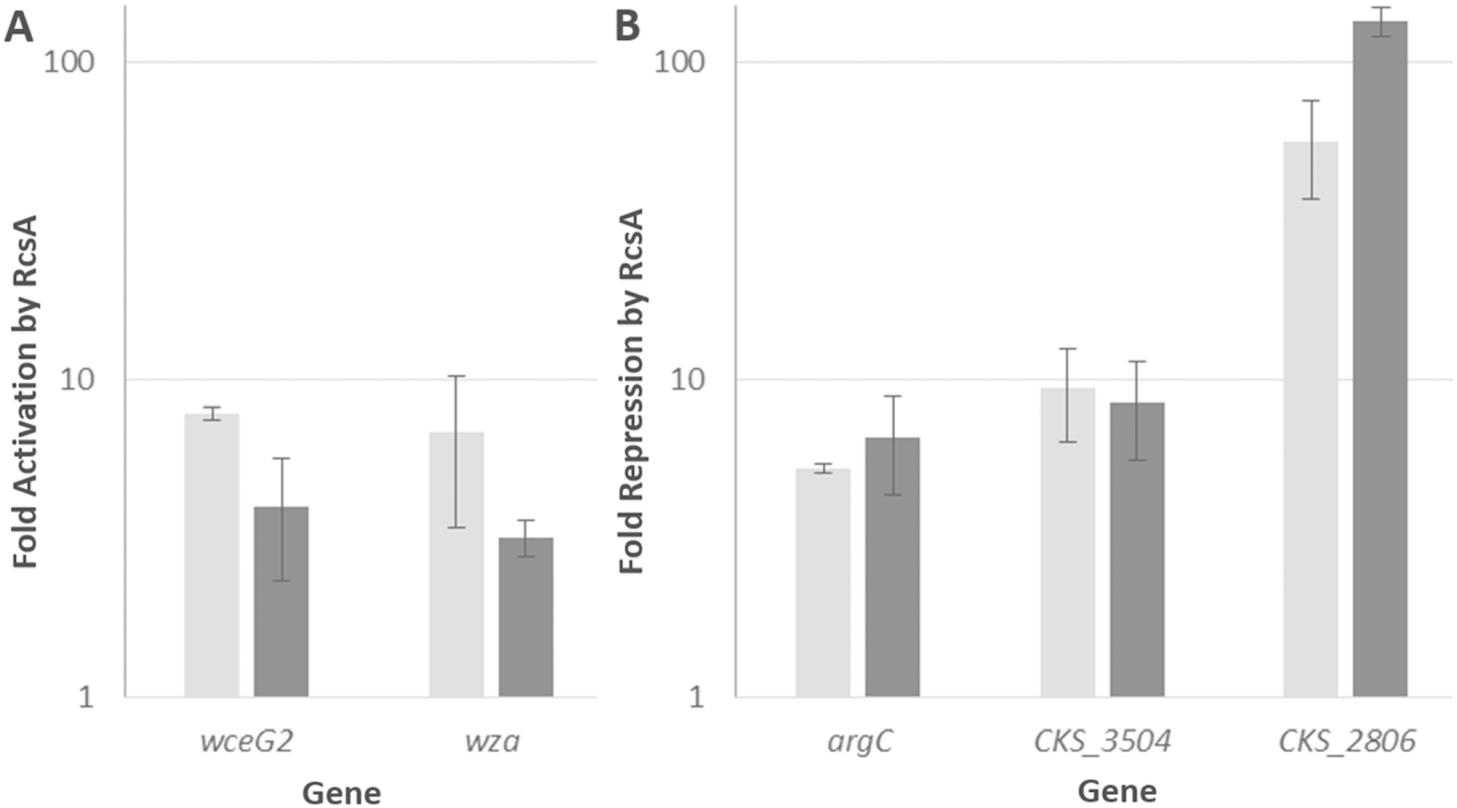
Validating transcriptional control of select genes by RcsA. Changes in gene expression were compared between the RNA-Seq analysis (light grey) and qRT-PCR assays (dark grey) for five genes regulated by RcsA. Y-axis represents the fold activation (panel A) or repression (panel B) on a logarithmic scale in the presence of RcsA. RNA-Seq results are averages of two experimental samples and qRT-PCR data represent two experimental samples analyzed in triplicate. Error bars were estimated using the sample standard error of the fold-change across the two independent biological replicates for both RNA-Seq and qRT-PCR.

**Fig 4:**
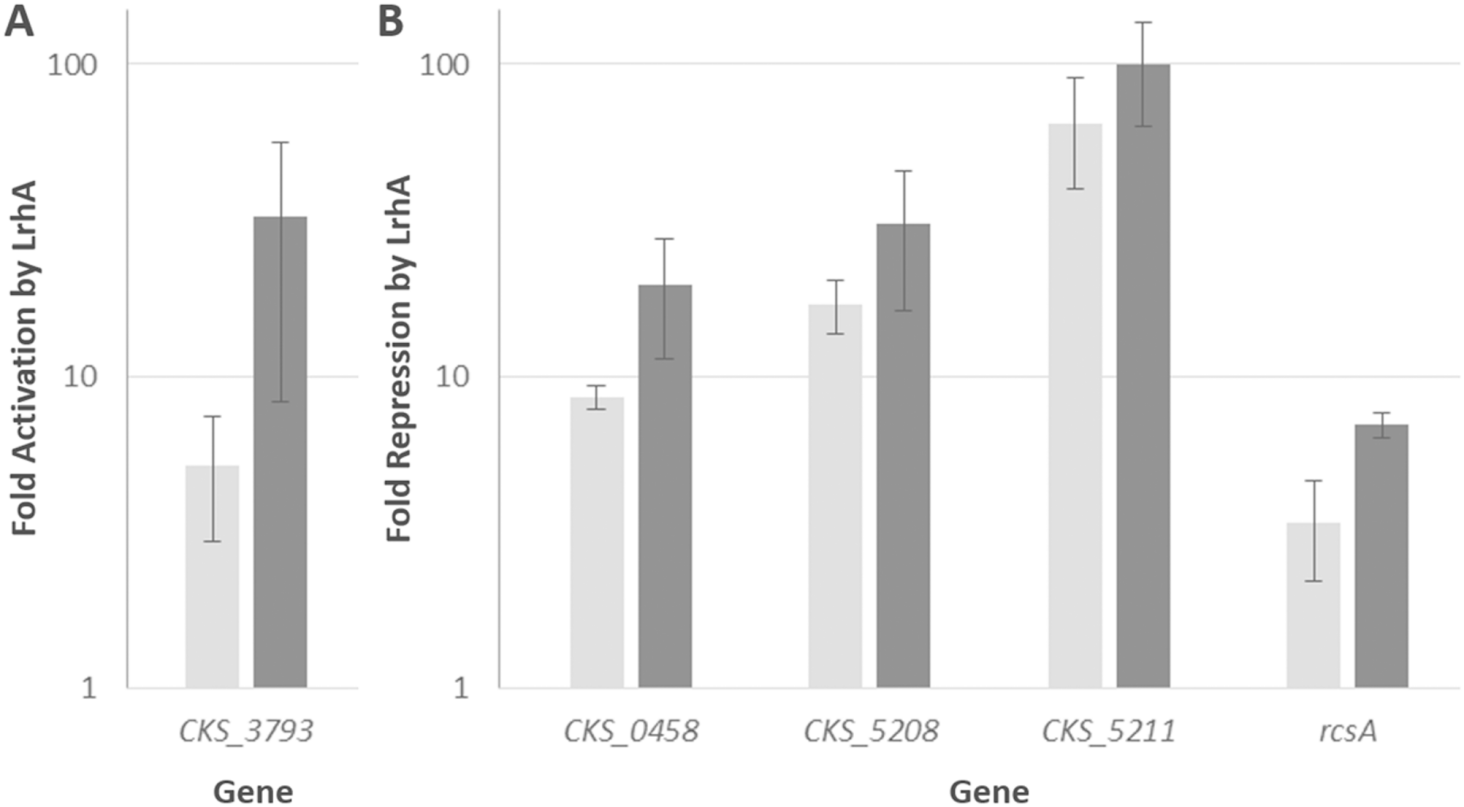
Validating transcriptional control of select genes by LrhA. Changes in gene expression were compared between the RNA-Seq analysis (light grey) and qRT-PCR assays (dark grey) for five genes regulated by LrhA. Y-axis represents the fold activation (panel A) or repression (panel B) on a logarithmic scale in the presence of LrhA. RNA-Seq results are averages of two experimental samples and qRT-PCR data represent two experimental samples analyzed in triplicate. Error bars were estimated using the sample standard error of the fold-change across the two independent biological replicates for both RNA-Seq and qRT-PCR.

Following the initial analysis of the RNA-Seq results, five target genes were selected from each of the putative RcsA and LrhA regulons to validate the RNA-Seq data via qRT-PCR. Some of the most highly regulated genes (Tables 2 and 3), which do not code for hypothetical proteins, were chosen for validation of the RNA-Seq data. The *rcsA* gene was also included in the analysis as its three-fold regulation by LrhA represented a potential feedback loop in the downstream QS system. The five genes tested for the RcsA regulon were: *wceG2*, *wza*, *argC*, CKS_3504, and CKS_2806. The five genes tested for the LrhA regulon were: CKS_3793, CKS_0458, CKS_5208, CKS_5211, and *rcsA*. The RNA-Seq data trends for the Δ*rcsA* and Δ*lrhA* strains were successfully validated by all five genes tested via qRT-PCR (Figs 3 and 4). Although there was variability in the absolute values of the fold changes between the RNA-Seq and qRT-PCR results, the regulation for a given gene shows similar trends (activation or repression) using both approaches. The qRT-PCR data also confirmed the initial observation from the RNA-Seq data that LrhA is repressing *rcsA* expression about three-fold. The interplay between these specific components of the *P*. *stewartii* QS system was thus further examined to build a more robust model of interactions.

### RcsA is controlled by LrhA

**Fig 5:**
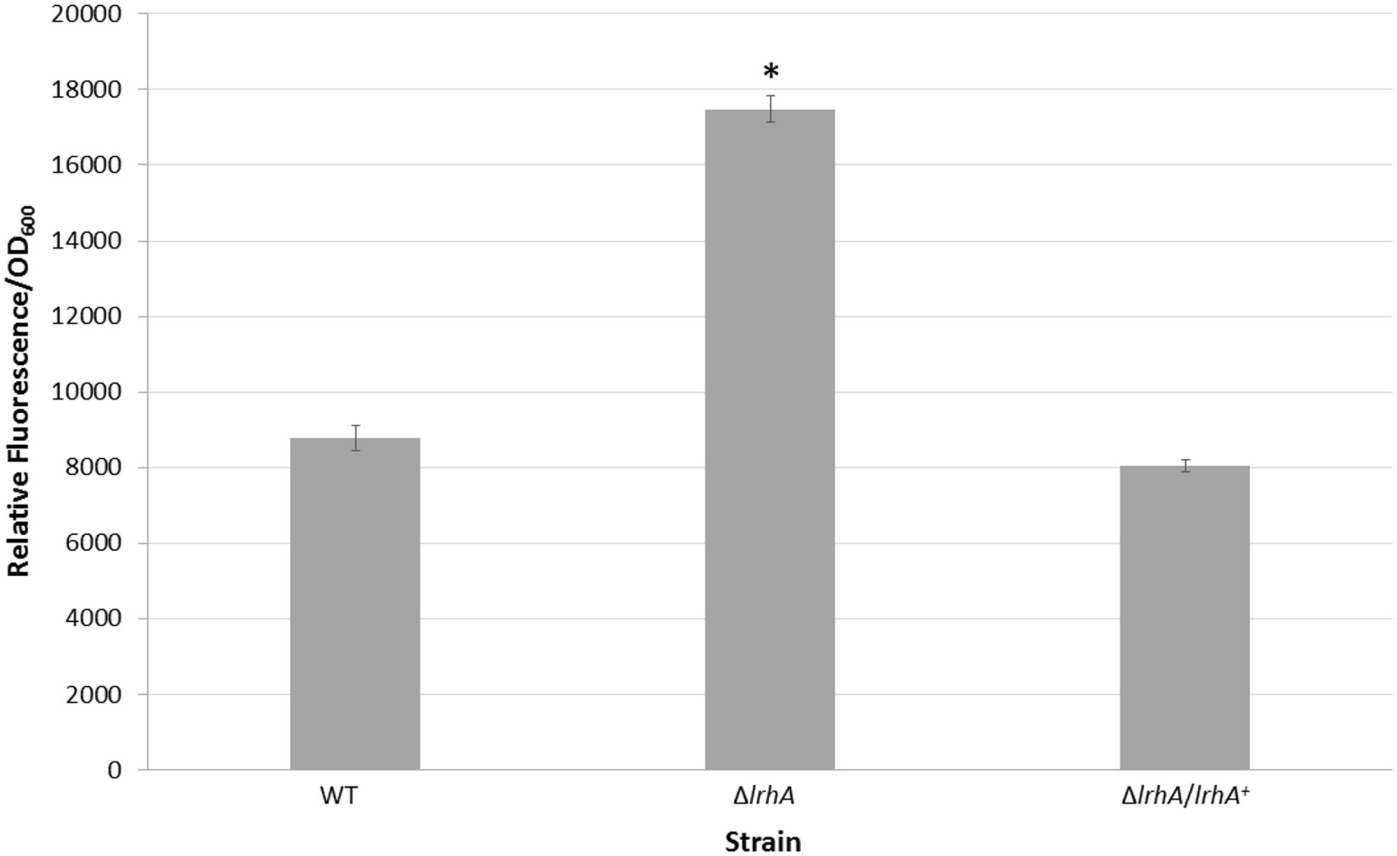
Expression from the *rcsA* promoter. A GFP reporter was used to measure levels of transcription from the *rcsA* promoter. Strains were grown to an OD_600_ of 0.5 and the average fluorescence/OD_600_ was measured. As indicated by an asterisk, expression from the Δ*lrhA* strain is significantly higher (*p*< 0.05) than either the wild-type strain or the Δ*lrhA/lrhA* ^*+*^ strain using a two-tailed homoscedastic Student’s t-test. This indicates that LrhA normally represses expression of *rcsA* in the wild-type strain. Data represents three experimental samples analyzed in triplicate. Error bars denote standard error.

A *rcsA* promoter-GFP reporter transcription fusion was used to analyze expression of *rcsA* in *P*. *stewartii* DC283 wild-type, Δ*lrhA* and Δ*lrhA/lrhA* ^*+*^ strains (Fig 5). These experiments were performed at an OD_600_ of 0.5, the same growth conditions used for the RNA-Seq and qRT-PCR analysis. The relative fluorescence units (RFU) produced by the Δ*lrhA* strain were significantly more (*p*< 0.05) than either the wild-type strain or the Δ*lrhA/lrhA* ^*+*^ strain, suggesting LrhA represses *rcsA* either directly or indirectly. The results of the GFP assays further verified that LrhA negatively regulates *rcsA*, confirming the existence of a previously unrecognized feedback loop in the regulatory circuitry downstream of EsaR in the QS network; it is now recognized that both EsaR and LrhA negatively regulate *rcsA*.

### RcsA and LrhA are involved in plant virulence

**Fig 6:**
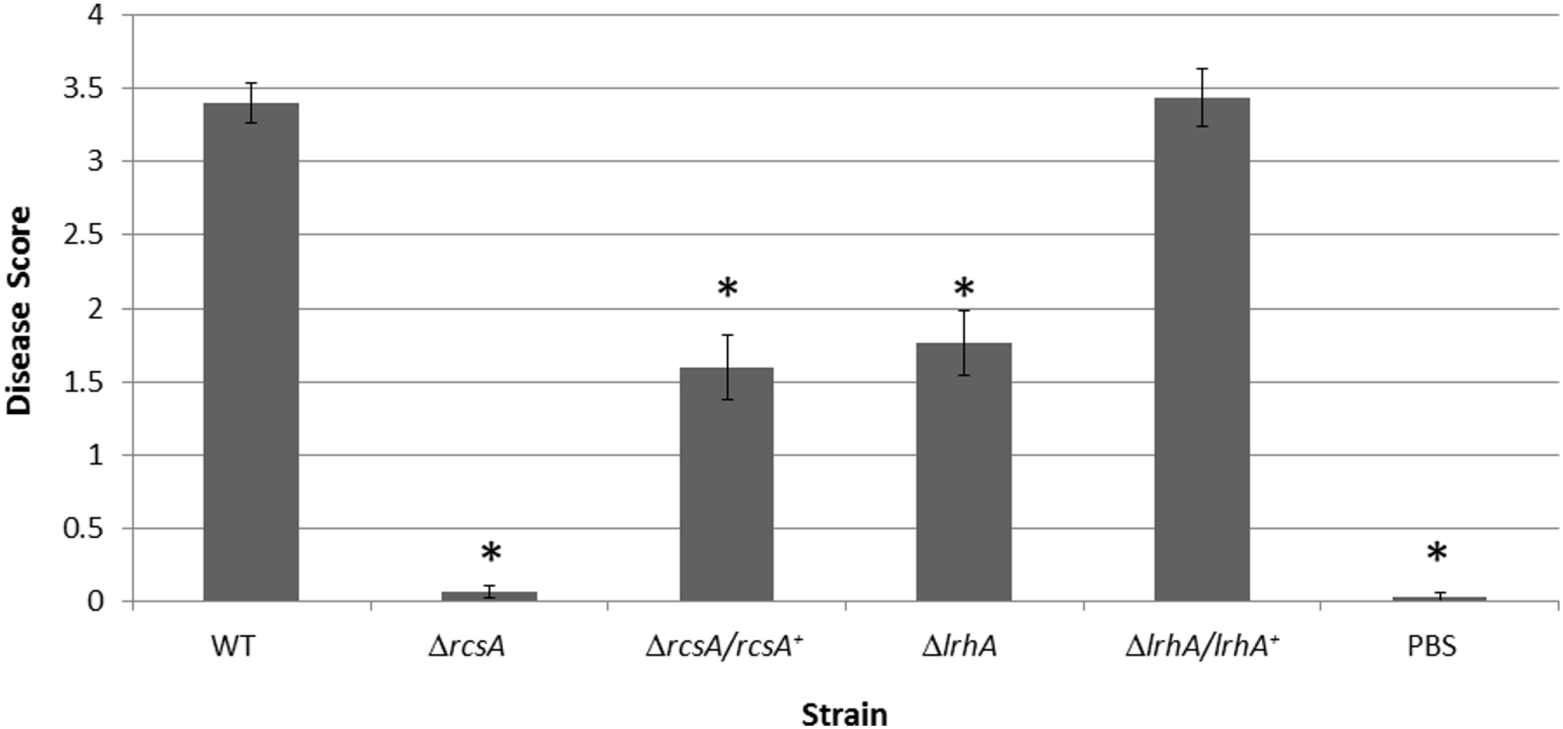
Plant assays testing the role of RcsA or LrhA in virulence. Data shown is the average score of disease for Day 12 of an infection assay performed with 15 plants inoculated with *P*. *stewartii* DC283 strains: wild type (WT), Δ*rcsA*, Δ*rcsA/rcsA* ^*+*^, Δ*lrhA*, Δ*lrhA/lrhA* ^*+*^, or PBS as a negative control. The asterisks (*) represent strains that are statistically significantly different (p< 0.05) from the wild-type strain using a two-tailed homoscedastic Student’s t-test. Error bars denote standard error.

The virulence of the Δ*rcsA* and Δ*lrhA* strains was compared to the wild type and respective complemented strains in a xylem-infection system. At day twelve post-infection, plants infected with the wild-type strain exhibit typical symptoms of the wilting stage and had an average disease severity score of ~3.5 while negative control plants infected with PBS were healthy with a score of ~0. The Δ*rcsA* strain also did not cause any severe disease symptoms with a score of ~0, while the Δ*lrhA* strain expressed an intermediate level of virulence with a score of ~1.75 (Fig 6). The Δ*rcsA/rcsaA* ^*+*^ strain partially restored the virulence of *P*. *stewartii*, whereas the Δ*lrhA/lrhA* ^*+*^ strain fully complemented virulence. Both RcsA and LrhA clearly play roles in the pathogenicity of *P*. *stewartii*.

## Discussion

Previous studies demonstrated that the quorum-sensing master regulator EsaR directly represses *rcsA* and directly activates *lrhA* transcription [15, 35]. As anticipated, phenotypic studies examining the impact of deletion of the genes encoding RcsA or LrhA resulted in noticeable effects on the production of capsule and the motility of *P*. *stewartii* cells, respectively. These important second-tier transcription factors downstream in the QS regulon were further examined for their role in global gene regulation via RNA-Seq analysis of gene expression in each of the two deletion strains compared with the wild-type strain. A qRT-PCR analysis confirmed that five genes for each deletion were regulated in the same manner shown by the RNA-Seq data. This allowed for increased confidence in the RNA-Seq data and the ability to draw more solid conclusions about the transcriptomes and the downstream network of gene regulation controlled by RcsA and LrhA.

Many of the most highly activated genes found in the RcsA regulon were related to capsule production: *wceG1*, *wceG2*, *wza*, *wzb*, *wzc*, *wceO*, *and wceF* (Table 2). Previous work has demonstrated that RcsA directly activates the promoters of the *cps* gene cluster in both *E*. *coli* and *P*. *stewartii* [4, 16, 19]. In *E*. *coli*, RcsA is one of two colonic acid capsular polysaccharide transcriptional activators, it is also self-activating, degraded by Lon-proteases, and requires RcsB to activate *cps* genes [16, 37]. Similarly, in *P*. *stewartii* RcsA has been shown to activate genes required for EPS production as well as to self-activate its own gene [16, 35]. The RNA-Seq data also revealed additional genes in the *P*. *stewartii* RcsA regulon that are directly or indirectly suppressed by RcsA. Specifically, RcsA repressed several genes in the *cys* and *arg* operons more than four-fold (Table 2). The *cys* genes are involved in sulfate activation, which leads to cysteine biogenesis in *E*. *coli* [38]. The genes *cysD* and *cysN* are in an operon and each encodes a subunit of the sulfate adenyltransferase complex [39, 40]. The *arg* genes are involved in arginine biosynthesis in *E*. *coli* [41, 42]. The three *arg* genes most regulated by RcsA are *argB* encoding acetylglutamate kinase, *argC* encoding N-acetyl-gamma-glutamylphosphate reductase, and *argI* encoding ornithine carbamoyltransferase. It is unclear why RcsA would repress the arginine and cysteine biosynthetic pathways and how this might relate to capsule production in *P*. *stewartii*.

*P*. *stewartii* LrhA is 77% identical at the amino acid level to its *E*. *coli* counterpart. In *E*. *coli*, LrhA is a LysR-type transcriptional factor negatively controlling motility, chemotaxis, flagellar biosynstheis [20] and type 1 fimbrial expression [43]. However, unlike *E*. *coli*, where LrhA highly represses some genes related to flagellar function and chemotaxis by approximately 3–80 fold [20], the *P*. *stewartii* RNA-Seq data only indicated a ~two- to three-fold level of repression. On the other hand, LrhA does repress expression of two putative fimbrial subunits (encoded by CKS_0458 and CKS_0459) more than four-fold in *P*. *stewartii* (Table 3 and Fig 4), while the *E*. *coli* K12 strain MG1655 genome (CP009685.1) does not contain homologues of these genes. Thus there are clear differences between the role of LrhA in *E*. *coli* and *P*. *stewartii*. Interestingly, the *P*. *stewartii* fimbrial subunit genes CKS_0458 and CKS_0459 were also previously found to be directly controlled by the master QS regulator EsaR [15]. This is another example of coordinated control of gene expression at more than one level in the *P*. *stewartii* QS regulatory network.

Other *P*. *stewartii* annotated genes regulated by LrhA include CKS_5208, encoding rhamnosyltransferase I subunit B (RhlB), which is repressed. This enzyme may be involved in surfactant production necessary to facilitate swarming motility. In addition, the gene most highly repressed by LrhA, CKS_5211, is located adjacent to CKS_5208 in the genome. Although originally annotated as a putative alpha/beta superfamily hydrolase/acyltransferase, CKS_5211 has very high homology with rhamnosyltransferase I subunit A (RhlA) of other sequenced strains of *Pantoea*. This further suggests an important role for LrhA in controlling surfactant production in *P*. *stewartii*. One annotated activated gene, CKS_3793, codes for cytochrome d ubiquinol oxidase subunit I, which presumably plays a role during aerobic respiration. The only other annotated gene found to be four-fold or more activated by LrhA was *wceO*, which is related to capsule synthesis and is also activated by RcsA, suggesting that its regulation is highly complex.

**Fig 7:**
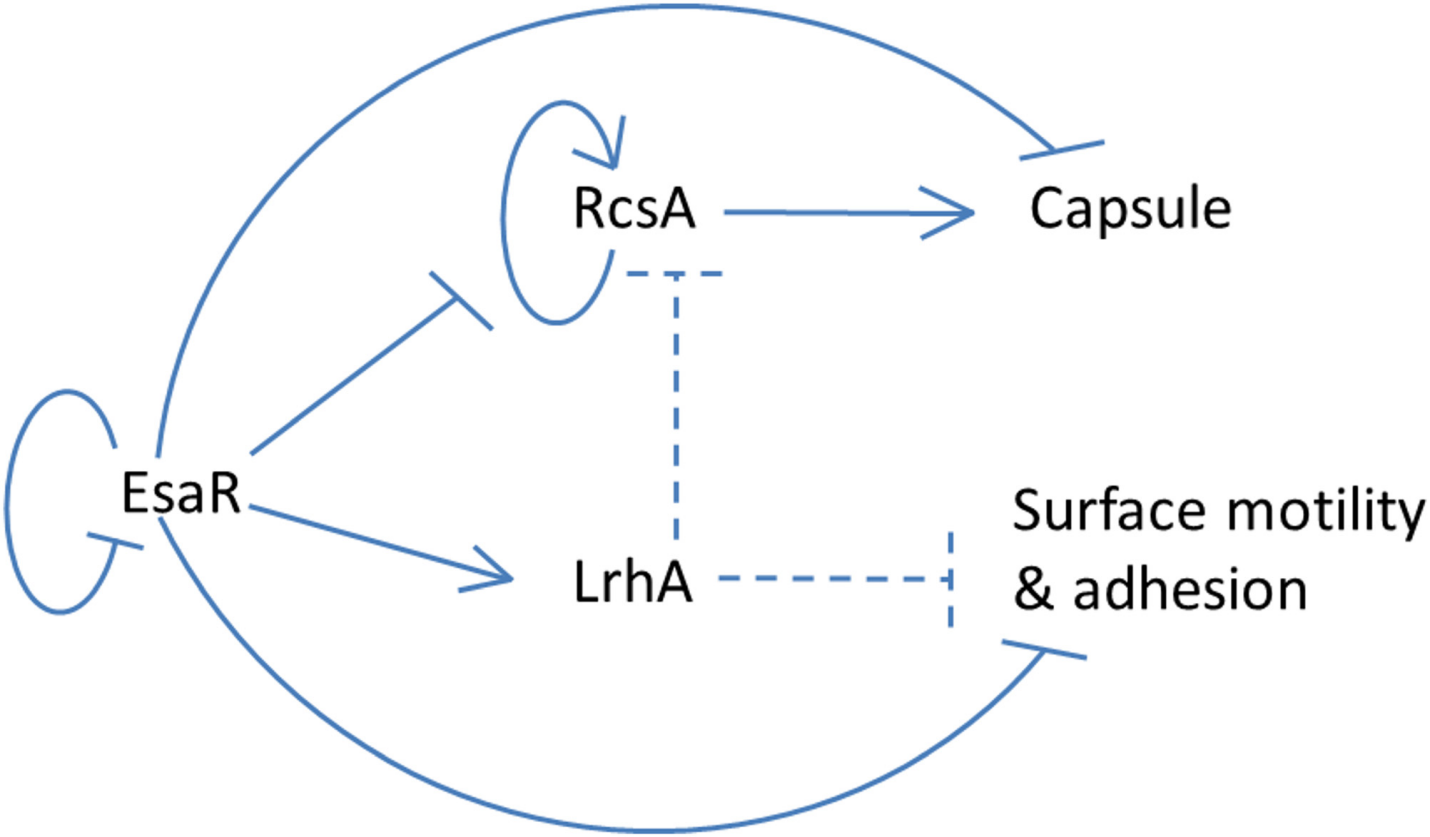
Model of the quorum-sensing regulatory network in *P*. *stewartii*. See the text for details. Solid lines indicate known direct regulatory control. Dashed lines indicate either direct or indirect control found in the present study. Arrows represent activation and T lines represent repression.

The RNA-Seq, qRT-PCR and transcriptional fusion experiments all confirmed the existence of a previously unknown feedback loop in the quorum-sensing network downstream of EsaR, with LrhA repressing RcsA. A developing model of this network suggests that there is tight coordinate control of key virulence factors in *P*. *stewartii* (Fig 7) [14, 15, 35] with the EsaR, RcsA, and LrhA proteins creating a coherent type three feed forward loop [44]. The repressive role exerted by EsaR on *rcsA* transcription appears to be reinforced by the negative regulation of *rcsA* exerted by the EsaR-activated gene product LrhA. Negative control of *rcsA* expression by LrhA likely helps repress capsule production in the bacterium until the correct temporal point in disease progression. Positive autoregulation of RcsA in *P*. *stewartii* [35] will result in higher levels of the protein that escape proteolysis by Lon [16], thereby reinforcing the signal to increase capsule production at high cell density [35]. Normally, QS tightly controls expression of *rcsA* so that the bacterium only produces high levels of capsule after migration to the xylem has occurred. If capsule is expressed too early, it can actually hinder the ability of *P*. *stewartii* to cause disease [4]. Alternatively, the Δ*rcsA* strain showed no sign of infection in the plant virulence assay. Thus if the bacterium is incapable of producing capsule it also cannot form a biofilm in the xylem. The complement of *rcsA* however only partially restored the virulence of the deletion strain, perhaps indicating how important precise fine-tuning of *rcsA* expression is to disease outcome. The Δ*lrhA* strain significantly decreased the virulence of *P*. *stewartii*, but not to the degree seen with the Δ*rcsA* strain. This may in part be due to the removal of one level of regulation of *rcsA*, but is also probably due to impacts on the adhesion and motility of the bacterium, an area of on-going research. Since RcsA controls capsule production in *P*. *stewartii* and capsule is a main virulence factor for the bacterium, it would make sense for it to be regulated at multiple points in the regulatory network to ensure that the bacteria successfully migrate to the xylem before capsule and biofilm production begins in the corn plant. The tight control of RcsA by QS and the downstream network of transcriptional regulators, including RcsA itself [35] and LrhA, help ensure the precise timing of disease progression.

## Supporting Information

S1 Table Primers used for strain construction. (DOCX)

S2 Table Primers used for qRT-PCR. (DOCX)

## References

[1] Roper MC. *Pantoea stewartii* subsp. *stewartii*: lessons learned from a xylem-dwelling pathogen of sweet corn. Mol Plant Pathol. 2011;12(7):628–37. 10.1111/j.1364-3703.2010.00698.x . 21726365 PMC6640275

[2] Esker PD, Nutter FW. Assessing the risk of Stewart's disease of corn through improved knowledge of the role of the corn flea beetle vector. Phytopathology. 2002;92(6):668–70. 10.1094/PHYTO.2002.92.6.668 . 18944266

[3] Braun EJ. Ultrastructural investigation of resistant and susceptible maize inbreds infected with *Erwinia stewartii* . Phytopathology. 1982;72(1):159–66.

[4] Minogue TD, Carlier AL, Koutsoudis MD, von Bodman SB. The cell density-dependent expression of stewartan exopolysaccharide in *Pantoea stewartii ssp*. *stewartii* is a function of EsaR-mediated repression of the *rcsA* gene. Mol Microbiol. 2005;56(1):189–203. Epub 2005/02/07. 10.1111/j.1365-2958.2004.04529.x . 15773989

[5] Fuqua WC, Winans SC, Greenberg EP. Quorum sensing in bacteria: the LuxR-LuxI family of cell density-responsive transcriptional regulators. J Bacteriol. 1994;176(2):269–75. 8288518 10.1128/jb.176.2.269-275.1994 PMC205046

[6] Joint I, Allan Downie J, Williams P. Bacterial conversations: talking, listening and eavesdropping. An introduction. Philos Trans R Soc Lond B Biol Sci. 2007;362(1483):1115–7. 10.1098/rstb.2007.2038 17360281 PMC2042524

[7] von Bodman SB, Willey JM, Diggle SP. Cell-cell communication in bacteria: united we stand. J Bacteriol. 2008;190(13):4377–91. Epub 2008/05/02. 10.1128/JB.00486-08 18456806 PMC2446813

[8] Whitehead NA, Barnard AM, Slater H, Simpson NJ, Salmond GP. Quorum-sensing in Gram-negative bacteria. FEMS Microbiol Rev. 2001;25(4):365–404. . 11524130 10.1111/j.1574-6976.2001.tb00583.x

[9] de Kievit TR, Iglewski BH. Bacterial quorum sensing in pathogenic relationships. Infect Immun. 2000;68(9):4839–49. 10948095 10.1128/iai.68.9.4839-4849.2000 PMC101676

[10] Beck von Bodman S, Farrand SK. Capsular polysaccharide biosynthesis and pathogenicity in *Erwinia stewartii* require induction by an N-acylhomoserine lactone autoinducer. J Bacteriol. 1995;177(17):5000–8. 7665477 10.1128/jb.177.17.5000-5008.1995 PMC177277

[11] von Bodman SB, Ball JK, Faini MA, Herrera CM, Minogue TD, Urbanowski ML, et al The quorum sensing negative regulators EsaR and ExpR(Ecc), homologues within the LuxR family, retain the ability to function as activators of transcription. J Bacteriol. 2003;185(23):7001–7. 14617666 10.1128/JB.185.23.7001-7007.2003 PMC262718

[12] von Bodman SB, Majerczak DR, Coplin DL. A negative regulator mediates quorum-sensing control of exopolysaccharide production in *Pantoea stewartii subsp*. *stewartii* . Proc Natl Acad Sci U S A. 1998;95(13):7687–92. 9636211 10.1073/pnas.95.13.7687 PMC22724

[13] Shong J, Huang YM, Bystroff C, Collins CH. Directed evolution of the quorum-sensing regulator EsaR for increased signal sensitivity. ACS Chem Biol. 2013;8(4):789–95. Epub 2013/02/06. 10.1021/cb3006402 23363022 PMC4478592

[14] Ramachandran R, Stevens AM. Proteomic analysis of the quorum-sensing regulon in *Pantoea stewartii* and identification of direct targets of EsaR. Appl Environ Microbiol. 2013;79(20):6244–52. Epub 2013/08/02. 10.1128/AEM.01744-13 23913428 PMC3811202

[15] Ramachandran R, Burke AK, Cormier G, Jensen RV, Stevens AM. Transcriptome-based analysis of the *Pantoea stewartii* quorum-sensing regulon and identification of EsaR direct targets. Appl Environ Microbiol. 2014;80(18):5790–800. 10.1128/AEM.01489-14 25015891 PMC4178595

[16] Ebel W, Trempy JE. *Escherichia coli* RcsA, a positive activator of colanic acid capsular polysaccharide synthesis, functions to activate its own expression. J Bacteriol. 1999;181(2):577–84. 9882673 10.1128/jb.181.2.577-584.1999 PMC93413

[17] Keenleyside WJ, Jayaratne P, MacLachlan PR, Whitfield C. The *rcsA* gene of *Escherichia coli* O9:K30:H12 is involved in the expression of the serotype-specific group I K (capsular) antigen. J Bacteriol. 1992;174(1):8–16. 1729226 10.1128/jb.174.1.8-16.1992 PMC205669

[18] Virlogeux I, Waxin H, Ecobichon C, Lee JO, Popoff MY. Characterization of the *rcsA* and *rcsB* genes from *Salmonella typhi*: *rcsB* through *tviA* is involved in regulation of Vi antigen synthesis. J Bacteriol. 1996;178(6):1691–8. 8626298 10.1128/jb.178.6.1691-1698.1996 PMC177855

[19] Carlier A, Burbank L, von Bodman SB. Identification and characterization of three novel EsaI/EsaR quorum-sensing controlled stewartan exopolysaccharide biosynthetic genes in *Pantoea stewartii ssp*. *stewartii* . Mol Microbiol. 2009;74(4):903–13. Epub 2009/10/15. 10.1111/j.1365-2958.2009.06906.x . 19843225

[20] Lehnen D, Blumer C, Polen T, Wackwitz B, Wendisch VF, Unden G. LrhA as a new transcriptional key regulator of flagella, motility and chemotaxis genes in *Escherichia coli* . Mol Microbiol. 2002;45(2):521–32. . 12123461 10.1046/j.1365-2958.2002.03032.x

[21] Herrera CM, Koutsoudis MD, Wang X, von Bodman SB. *Pantoea stewartii* subsp. *stewartii* exhibits surface motility, which is a critical aspect of Stewart's wilt disease development on maize. Mol Plant Microbe Interact. 2008;21(10):1359–70. 10.1094/MPMI-21-10-1359 . 18785831

[22] Francez-Charlot A, Laugel B, Van Gemert A, Dubarry N, Wiorowski F, Castanie-Cornet MP, et al RcsCDB His-Asp phosphorelay system negatively regulates the *flhDC* operon in *Escherichia coli* . Mol Microbiol. 2003;49(3):823–32. . 12864862 10.1046/j.1365-2958.2003.03601.x

[23] Clemmer KM, Rather PN. Regulation of *flhDC* expression in *Proteus mirabilis* . Res Microbiol. 2007;158(3):295–302. 10.1016/j.resmic.2006.11.010 . 17320355

[24] Wang Q, Zhao Y, McClelland M, Harshey RM. The RcsCDB signaling system and swarming motility in *Salmonella enterica* serovar *typhimurium*: dual regulation of flagellar and SPI-2 virulence genes. J Bacteriol. 2007;189(23):8447–57. 10.1128/JB.01198-07 17905992 PMC2168921

[25] Kvitko BH, Bruckbauer S, Prucha J, McMillan I, Breland EJ, Lehman S, et al A simple method for construction of *pir+ Enterobacteria*l hosts for maintenance of R6K replicon plasmids. BMC Res Notes. 2012;5:157 10.1186/1756-0500-5-157 22433797 PMC3338088

[26] Dolph PJ, Majerczak DR, Coplin DL. Characterization of a gene cluster for exopolysaccharide biosynthesis and virulence in *Erwinia stewartii* . J Bacteriol. 1988;170(2):865–71. 2828330 10.1128/jb.170.2.865-871.1988 PMC210734

[27] Herrero M, de Lorenzo V, Timmis KN. Transposon vectors containing non-antibiotic resistance selection markers for cloning and stable chromosomal insertion of foreign genes in Gram-negative bacteria. J Bacteriol. 1990;172(11):6557–67. 2172216 10.1128/jb.172.11.6557-6567.1990 PMC526845

[28] Stabb EV, Ruby EG. RP4-based plasmids for conjugation between *Escherichia coli* and members of the *Vibrionaceae* . Methods Enzymol. 2002;358:413–26. . 12474404 10.1016/s0076-6879(02)58106-4

[29] Kaniga K, Delor I, Cornelis GR. A wide-host-range suicide vector for improving reverse genetics in gram-negative bacteria: inactivation of the *blaA* gene of *Yersinia enterocolitica* . Gene. 1991;109(1):137–41. . 1756974 10.1016/0378-1119(91)90599-7

[30] Choi KH, Gaynor JB, White KG, Lopez C, Bosio CM, Karkhoff-Schweizer RR, et al A Tn7-based broad-range bacterial cloning and expression system. Nat Methods. 2005;2(6):443–8. 10.1038/nmeth765 . 15908923

[31] Labes M, Puhler A, Simon R. A new family of Rsf1010-derived expression and Lac-fusion broad-host-range vectors for Gram-negative bacteria. Gene. 1990;89(1):37–46. 10.1016/0378-1119(90)90203-4 2115488

[32] Burbank L, Roper MC. OxyR and SoxR modulate the inducible oxidative stress response and are implicated during different stages of infection for the bacterial phytopathogen *Pantoea stewartii* subsp. *stewartii* . Mol Plant Microbe Interact. 2014;27(5):479–90. 10.1094/MPMI-11-13-0348-R . 24450773

[33] Anders S, Huber W. Differential expression analysis for sequence count data. Genome Biol. 2010;11(10):R106 10.1186/gb-2010-11-10-r106 20979621 PMC3218662

[34] Pfaffl MW. A new mathematical model for relative quantification in real-time RT–PCR. Nucleic Acids Res. 2001;29(9):e45 10.1093/nar/29.9.e45 11328886 PMC55695

[35] Carlier AL, von Bodman SB. The *rcsA* promoter of *Pantoea stewartii* subsp. *stewartii* features a low-level constitutive promoter and an EsaR quorum-sensing-regulated promoter. J Bacteriol. 2006;188(12):4581–4. 10.1128/JB.00211-06 16740966 PMC1482964

[36] Miller WG, Leveau JH, Lindow SE. Improved *gfp* and *inaZ* broad-host-range promoter-probe vectors. Mol Plant Microbe Interact. 2000;13(11):1243–50. 10.1094/MPMI.2000.13.11.1243 . 11059491

[37] Hagiwara D, Sugiura M, Oshima T, Mori H, Aiba H, Yamashino T, et al Genome-wide analyses revealing a signaling network of the RcsC-YojN-RcsB phosphorelay system in *Escherichia coli* . J Bacteriol. 2003;185(19):5735–46. 13129944 10.1128/JB.185.19.5735-5746.2003 PMC193970

[38] Awano N, Wada M, Mori H, Nakamori S, Takagi H. Identification and functional analysis of *Escherichia coli* cysteine desulfhydrases. Appl Environ Microbiol. 2005;71(7):4149–52. 10.1128/AEM.71.7.4149-4152.2005 16000837 PMC1169034

[39] Malo MS, Loughlin RE. Promoter elements and regulation of expression of the *cysD* gene of *Escherichia coli* K-12. Gene. 1990;87(1):127–31. . 2185135 10.1016/0378-1119(90)90504-k

[40] Leyh TS, Taylor JC, Markham GD. The sulfate activation locus of *Escherichia coli* K12: cloning, genetic, and enzymatic characterization. J Biol Chem. 1988;263(5):2409–16. . 2828368

[41] Ludovice M, Martin JF, Carrachas P, Liras P. Characterization of the *Streptomyces clavuligerus argC* gene encoding N-acetylglutamyl-phosphate reductase: expression in *Streptomyces lividan*s and effect on clavulanic acid production. J Bacteriol. 1992;174(14):4606–13. 1339424 10.1128/jb.174.14.4606-4613.1992 PMC206255

[42] Parsot C, Boyen A, Cohen GN, Glansdorff N. Nucleotide sequence of *Escherichia coli argB* and *argC* genes: comparison of N-acetylglutamate kinase and N-acetylglutamate-gamma-semialdehyde dehydrogenase with homologous and analogous enzymes. Gene. 1988;68(2):275–83. . 2851495 10.1016/0378-1119(88)90030-3

[43] Blumer C, Kleefeld A, Lehnen D, Heintz M, Dobrindt U, Nagy G, et al Regulation of type 1 fimbriae synthesis and biofilm formation by the transcriptional regulator LrhA of *Escherichia coli* . Microbiology. 2005;151(Pt 10):3287–98. 10.1099/mic.0.28098-0 . 16207912

[44] Alon U. Network motifs: theory and experimental approaches. Nat Rev Genet. 2007;8(6):450–61. 10.1038/nrg2102 . 17510665

[45] Grant SG, Jessee J, Bloom FR, Hanahan D. Differential plasmid rescue from transgenic mouse DNAs into *Escherichia coli* methylation-restriction mutants. Proc Natl Acad Sci U S A. 1990;87(12):4645–9. 2162051 10.1073/pnas.87.12.4645 PMC54173

[46] Raleigh EA, Murray NE, Revel H, Blumenthal RM, Westaway D, Reith AD, et al McrA and McrB restriction phenotypes of some *E*. *coli* strains and implications for gene cloning. Nucleic Acids Res. 1988;16(4):1563–75. 2831502 10.1093/nar/16.4.1563 PMC336335

