# Supplementary material for: Analyzing the Transcriptomes of Two Quorum-Sensing Controlled Transcription Factors, RcsA and LrhA, Important for Pantoea stewartii Virulence
Source: PLoS One. 2015 Dec 23;10(12):e0145358. doi: 10.1371/journal.pone.0145358 (PMC4689408; doi:10.1371/journal.pone.0145358)
Supplement: S1 Table — (DOCX) [file pone.0145358.s001.docx]

**S1 Table: Primers used for strain construction**

| **Primers** | **Sequence (5’ to 3’)** | **Purpose** |
| --- | --- | --- |
| **Deletion construction** | | |
| LrhA-UPF | GTCGACATTGTCCAGTTTGCCGG | Amplify 1 kb region upstream of *lrhA* |
| LrhA-UPR | AGTGGAATATAGGCGGCCGCCTTCACTTATTAGAG |  |
| LrhA-DNF | GCGGCCGCCTATATTCCACTATCCCGTCTTC | Amplify 1 kb region downstream of *lrhA* |
| LrhA-DNR | GGATCCCCAATGCGCACCAG |  |
| LrhA-1kbUPF-attB1 | GGGGACAAGTTTGTACAAAAAAGCAGGCTGTCGACGTTTGCCGGATTTATCAATTTG | Amplify 2 kb deletion fragment of *lrhA* with flanking *attB* sites |
| LrhA-1kbDNR-attB2 | GGGGACCACTTTGTACAAGAAAGCTGGGTGGATCCGCGCACCAGATAAACCAGGC |  |
| UP-LrhA-SeqF | GTATGACAGACCCATTTACCCCG | Screen/sequence mutants for *lrhA* deletion |
| IN-LrhA-SeqF | GCGATCCCTCTGGTATTGCTGG |  |
| DN-LrhA-SeqR | GCCCTGTTGGCCAGAGTATG |  |
| RcsA-UPF | GTCGACATCCTTCAACGGTCATTTGTG | Amplify 1 kb region upstream of *rcsA* |
| RcsA-UPR | AGTGGAATATAGGCGGCCGCCTCACCAATTTGTTATC |  |
| RcsA-DNF | GCGGCCGCCTATATTCCACTCCTCACAGAACTG | Amplify 1 kb region downstream of *rcsA* |
| RcsA-DNR | GGATCCGATAGCGCCTTCAAGC |  |
| RcsA-1kbUPF-attB1 | GGGGACAAGTTTGTACAAAAAAGCAGGCTGTCGACCAACGGTCATTTGTGGCTTATC | Amplify 2 kb deletion fragment of *rcsA* with flanking *attB* sites |
| RcsA-1kbDNR-attB2 | GGGGACCACTTTGTACAAGAAAGCTGGGTGGATCCGCCTTCAAGCACCGAACCGAC |  |
| UP-RcsA-SeqF | TACCCGATGTGGATTCGACGCC | Screen/sequence of mutants for *rcsA* deletion |
| IN-RcsA-SeqF | CCAGCCCTGAGGAAATACGCGG |  |
| DN-RcsA-SeqR | TTGAAATCATCCATCTCACCAATACGCGC |  |
| **Chromosomal complementation construction** | | |
| EcoRI-PlrhA-F | GAATTCTGCACAATGTACTCTCCTCACG | Amplify promoter and coding region of *lrhA,* and screen conjugants |
| XhoI-LrhA-R | CTCGAGCTATTACTCTTCATCGTCCAGCAG |  |
| SpeI-RcsA-F | ACTAGTGAAATTCACAACTATCCGGGCATTTTTC | Amplify promoter and coding region of *rcsA,* and screen conjugants |
| SacI-RcsA-R | GAGCTCCTATCTTACGTTGACGTAAATACCAG |  |
| *glmS* downstream | TCTCTGATAAGCACCATGCCCTGT | PCR screen and sequence for validation of chromosomal insertion [32] |
| *glmS* intergenic region rev | TACGGTGCTACGCATCAGTGTCAT |  |
| **GFP plasmid construction** | | |
| TFRcsAFwdEcoRI | GAATTGGAAATTCACAACTATCCGGGCATTTTTC | Primers used for GFP transcriptional fusion construction |
| TFRcsARevKpnI | GGTACCGTTAGCGACCCTCACCAATTTGTTATCC |  |
