# Supplementary material for: Analyzing the Transcriptomes of Two Quorum-Sensing Controlled Transcription Factors, RcsA and LrhA, Important for Pantoea stewartii Virulence
Source: PLoS One. 2015 Dec 23;10(12):e0145358. doi: 10.1371/journal.pone.0145358 (PMC4689408; doi:10.1371/journal.pone.0145358)
Supplement: S2 Table — (DOCX) [file pone.0145358.s002.docx]

**S2 Table: Primers used for qRT-PCR**

| **Gene** | **Primer** | **Annealing Temp** | **Sequence 5’ to 3’** |
| --- | --- | --- | --- |
| **Genes validated for RcsA regulon** | | | |
| ***argC*** | Cloning-Forward | 55°C | ATCGTTGGTGCCAGTGGTTACG |
|  | Cloning-Reverse |  | AGGGAAACCGAAACGAATATTAAGGCAC |
|  | RT-PCR Forward | 62°C | GTGGAGCAGGGCGCAAA |
|  | RT-PCR Reverse |  | AAATACCGTAAGGCTGCAGACTGA |
| **CKS_2806** | Cloning-Forward | 55°C | GAACGCGGTCTGGAACGG |
|  | Cloning-Reverse |  | TCAGGCAATGCGTTGGGTG |
|  | RT-PCR Forward | 64°C | GCGAAGGCCAGAATGTTGACA |
|  | RT-PCR Reverse |  | GCTCACGCGACGTGTTACG |
| **CKS_3504** | Cloning-Forward | 55°C | ATGCCAGCCGAAACATCTGCAGCA |
|  | Cloning-Reverse |  | TGCCGGGGAGACTGAATGGG |
|  | RT-PCR Forward | 64°C | GCGTTTTTCGTGCCATGGA |
|  | RT-PCR Reverse |  | CACTTTGCCCTGGGTGATCA |
| ***wceG2*** | Previously published [15] | | |
| ***wza*** | Cloning-Forward | 55°C | ATGATTACAATGAAAATGAAGATGATACCTGTTTTGG |
|  | Cloning-Reverse |  | TTAGTTCGACCAGTTGCGGATGC |
|  | RT-PCR Forward | 62°C | gcgaacagcgcgtgtca |
|  | RT-PCR Reverse |  | ATGGTTTTGGCTCAGAT |
| **Genes validated for LrhA regulon** | | | |
| **CKS0458** | Previously published [15] | | |
| **CKS_3793** | Cloning-Forward | 57°C | ATGCTAGATATCGTCGAACTGTC |
|  | Cloning-Reverse |  | TCTGTTCATGGTGATAGCGC |
|  | RT-PCR Forward | 64°C | CCTTTGTGGGCCTGTTCTTTTT |
|  | RT-PCR Reverse |  | ACCGCCAGATGCTGCACTT |
| **CKS_5208** | Cloning-Forward | 57°C | ATGAAAGTGATTATTGGCGCAC |
|  | Cloning-Reverse |  | TCTGTGGGTTTGATTAGCCAG |
|  | RT-PCR Forward | 60°C | TCAATAACGAACCGCAGTCGAT |
|  | RT-PCR Reverse |  | AATCCCTCGCGCGCTTT |
| **CKS_5211** | Cloning-Forward | 57°C | ATGAGTCACGGTTATTCTGTAGTAAAC |
|  | Cloning-Reverse |  | TCAACAAAGCCTACCACAGC |
|  | RT-PCR Forward | 60°C | CATTACGTTTGATCTTCCGTTCTCA |
|  | RT-PCR Reverse |  | GACGATATGGCAGCCCTGTTC |
| ***rcsA*** | Cloning-Forward | 55°C | ACTAGTGAAATTCACAACTATCCGGGCATTTTTC |
|  | Cloning-Reverse |  | GAGCTCCTATCTTACGTTGACGTAAATACCAG |
|  | RT-PCR Forward | 60°C | AGCGGAAAATTAAAACGCACAAC |
|  | RT-PCR Reverse |  | CAGAGGTCACGTTATCGGTTAAGC |
| **Gene used for normalization of samples** | | | |
| **16S rRNA** | Previously published [15] | | |
